# Supplementary material for: Concurrent Inhibition of Akt and ERK Using TIC-10 Can Overcome Venetoclax Resistance in Mantle Cell Lymphoma
Source: Cancers (Basel). 2023 Jan 13;15(2):510. doi: 10.3390/cancers15020510 (PMC9856512; doi:10.3390/cancers15020510)
Supplement: Supplementary file 1 [file cancers-15-00510-s001.zip › cancers-2138618-supplementary.pdf]

Supplementary Materials for

Concurrent inhibition of Akt and ERK using TIC-10 can overcome venetoclax resistance in mantle cell lymphoma

This PDF file includes:

Figures. S1 to S2
Tables S1 to S5

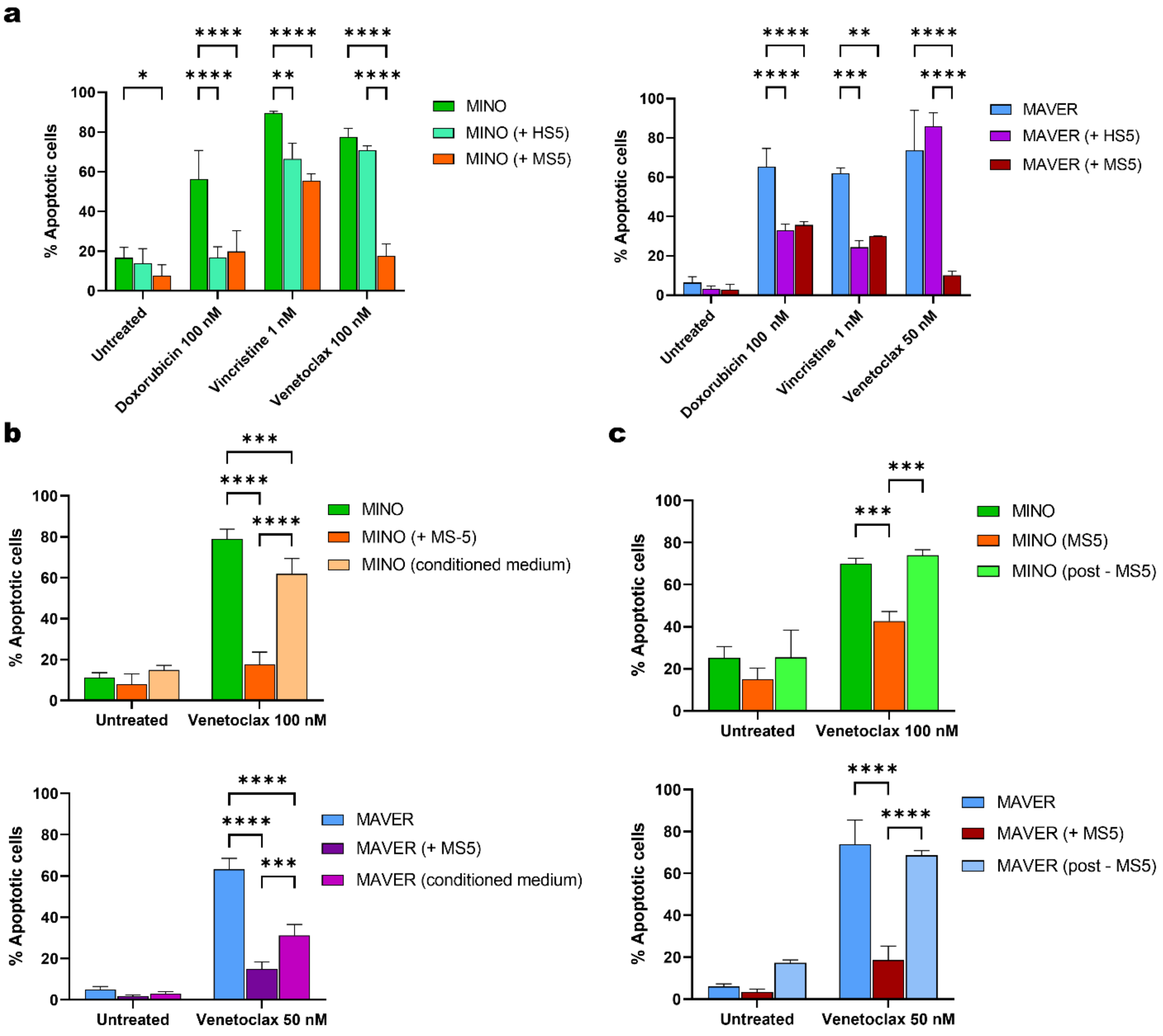

Figure S1. A) Bar charts with the apoptotic response of MINO (left) and MAVER (right) in monoculture and co-culture with HS-5 and MS-5, following treatment with doxorubicin vincristine and venetoclax for 48 hours. Data are shown as mean + SD (n =3). B) Bar charts of the measured apoptotic response of MINO (upper) and MAVER (lower) to venetoclax treatment in monoculture, co-culture with MS-5 and monoculture using MS-5-conditioned medium. Data are shown as mean + SD (n = 3). C) Bar charts of the mean apoptotic response of MINO (upper) and MAVER (lower) to venetoclax treatment in monoculture, co-culture with MS-5 and monoculture following a 48 hour co-culture with MS-5. Data are shown as mean + SD (n = 3)

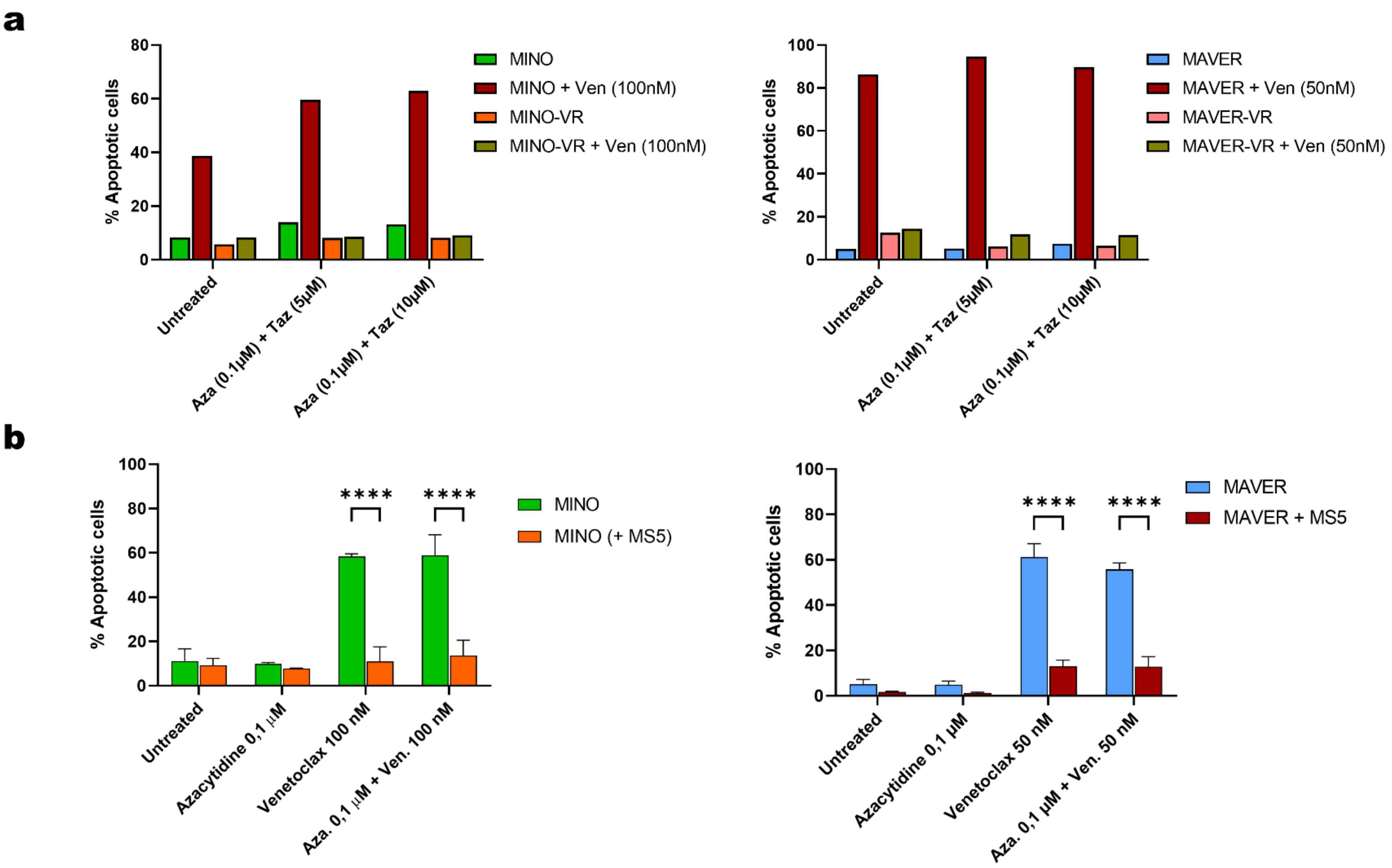

Figure S2. A) Bar charts with the apoptotic response of MINO/MINO-VR (left) and MAVER/MAVER-VR (right) after being treated with the epigenetic drugs 5-azacytidine (Aza) and Tazemetostat (Taz) in two different combination doses for 48 hours, followed by treatment with either nothing or venetoclax. Data are shown as mean + SD (n =3). B) Bar charts of the measured apoptotic response of MINO (left) and MAVER (right) in co-culture with MS-5 treated with Azacytidine prior to the treatment with venetoclax. Data are shown as mean + SD (n = 3).

Table S1.
Primer sequences of all primers used in the study.

**Gene**	**Forward primer**	**Reverse primer**
SF3A1	5´- TCCATCCGTGAGAAGCAGAGC - 3´	5´- TCTGGATCTCCTCCTCACCG - 3´
PUM1	5´- CATGCCAGGTTATCCGGTGT - 3´	5´- GCGCCTGCATTCACTACAAG - 3´
BCL2	5´- ACGACTTCTCCCGCCGCTAC- 3´	5´- CACCACCGTGGCAAAGCGT- 3´
MCL1	5´- CAAAGAGGCTGGGATGGGTT- 3´	5´- GCAACACCTGCAAAAGCCAG- 3´
BCL-XL	5´- GCAGGTATTGGTGAGTCGGA- 3´	5´- CCACAAAAGTATCCCAGCCG- 3´
BIM	5´- GGCCCCTACCTCCCTACA- 3´	5´- GGGGTTTGTGTTGATTTGTCA- 3´
NOXA	5´- GAGTGTGCTACTCAACTCAG- 3´	5´- CCTTGGAAACGGAAGATGGA- 3´
PUMA	5´- GACGACCTCAACGCACAGTA- 3´	5´- CTAATTGGGCTCCATCTCG- 3´
ACTB (Acce*SssI*ble)	5´- AGAGGGGGTAAAAAAATGTTGTAT - 3´	5´- TCGAACCATAAAAAACAACTTTC -3´
GRP78 (Acce*SssI*ble)	5´- AGATAGTTGTTGAATTAATGGGATT - 3´	5´- CCCCCCAACTAATTCATTAACTA -3´

Table S2
Calculated IC_{50} values for the MCL-1 inhibitor S63845 and the BCL-XL inhibitor WEHI-539 in the parental and venetoclax-resistant (VR) MINO and MAVER cells.

**Cell line**	**S63845 IC_{50} (μM)**	**WEHI-539 IC_{50} (μM)**
MINO	0.13	0.11
MINO-VR	0.89	104.8
MAVER	4.31	294.1
MAVER-VR	0.96	104.2

Table S3.

Overview of the 21 common mutations between venetoclax resistant MINO-VR and MAVER-VR, when comparing their genetic profile to the paternal MINO and MAVER, and after substraction of the genetic aberrations acquired during prolonged culture (MINO-LT and MAVER-LT cell lines).

Location	Allele	Consequence	SYMBOL	Gene	cDNA_position
4:73139975-73139975	A	stop_gained	ANKRD17	ENSG00000132466	2771
15:49931287-49931287	A	stop_gained	ATP8B4	ENSG00000104043	1627
8:112314512-112314512	T	stop_gained	CSMD3	ENSG00000164796	7551
11:108513912-108513912	T	stop_gained	EXPH5	ENSG00000110723	1827
16:31397595-31397595	A	splice_acceptor_variant	ITGAD	ENSG00000156886	-
3:66500405-66500405	A	start_lost	LRIG1	ENSG00000144749	618
X:71136596-71136596	T	stop_gained	MED12	ENSG00000184634	5500
10:128104037-128104037	T	frameshift_variant	MKI67	ENSG00000148773	8216-8217
11:102791332-102791332	G	splice_donor_variant	MMP1	ENSG00000196611	-
11:118262936-118262936	A	stop_gained	MPZL2	ENSG00000149573	364
5:36953698-36953698	A	start_lost	NIPBL	ENSG00000164190	491
7:83155605-83155605	A	frameshift_variant	PCLO	ENSG00000186472	1327-1328
10:3082387-3082387	C	splice_acceptor_variant	PFKP	ENSG00000067057	-
16:88733746-88733746	TGTGATGG	splice_acceptor_variant	PIEZO1	ENSG00000103335	-
8:56167337-56167337	A	stop_gained	PLAG1	ENSG00000181690	883
22:26292873-26292873	T	stop_gained	SEZ6L	ENSG00000100095	770
6:169248782-169248782	A	stop_gained	THBS2	ENSG00000186340	389
17:7676195-7676196	-	frameshift_variant	TP53	ENSG00000141510	315
9:137200363-137200363	A	stop_gained	TPRN	ENSG00000176058	379
5:124744570-124744598	-	frameshift_variant	ZNF608	ENSG00000168916	1020-1047
19:36819933-36819933	T	stop_gained	ZNF790	ENSG00000197863	535

Table S4.

Overview of the chromatin accessibility (ΔAcc.) and DNA methylation (ΔMeth.) changes between the venetoclax-resistant cell lines (MINO-VR, MAVER-VR) and their paternal counterpart, in the promoter area of *BCL2*, *MCL1* and *BCL2L1* (coding for BCL-XL). Significant changes that potentially can lead to gene upregulation are marked in green, while changes that can lead to gene downregulation are marked in red.

Illumina probe ID	UCSC_RefGene_Name	UCSC_RefGene_Group	ΔAcc. MINO-VR	ΔMeth. MINO-VR	ΔAcc. MAVER-VR	ΔMeth. MAVER-VR

cg05891614	BCL2	3'UTR	-0,092	0,139	-0,152	0,027
cg21984711	BCL2	3'UTR	-0,103	0,052	0,037	-0,011
cg17192381	BCL2	3'UTR	-0,004	-0,105	0,123	-0,040
cg20057935	BCL2	Body	0,003	0,025	-0,028	-0,012
cg11655407	BCL2	Body	-0,027	0,158	0,058	0,235
cg22177062	BCL2	Body	-0,020	0,575	0,139	0,107
cg01282639	BCL2	Body	-0,044	0,232	0,020	-0,013
cg27311011	BCL2	Body	0,006	0,040	-0,031	0,005
cg09898122	BCL2	Body	-0,056	0,042	-0,114	-0,022
cg06631916	BCL2	Body	0,005	0,070	0,008	0,017
cg16156297	BCL2	Body	-0,033	0,002	-0,044	-0,013
cg02288628	BCL2	Body	-0,090	0,016	-0,001	-0,039
cg20593471	BCL2	Body	-0,065	-0,019	-0,016	-0,023
cg08633051	BCL2	Body	-0,076	0,179	0,082	-0,106
cg09698619	BCL2	Body	0,029	-0,151	0,047	0,320
cg19768034	BCL2	Body	-0,026	0,190	-0,013	0,226
cg17079083	BCL2	Body	-0,035	0,165	0,032	0,051
cg12696761	BCL2	Body	-0,061	0,109	-0,014	0,120
cg01100915	BCL2	Body	-0,047	0,076	-0,033	0,072
cg12395867	BCL2	Body	-0,062	0,164	-0,029	0,136
cg07788351	BCL2	Body	-0,055	0,133	-0,036	0,273

cg13042130	BCL2	Body	-0,068	0,180	-0,034	0,256
cg14833556	BCL2	Body	-0,021	-0,101	-0,014	0,083
cg26856497	BCL2	Body	0,054	0,010	0,031	-0,075
cg01108734	BCL2	Body	-0,007	0,048	0,065	-0,026
cg19875106	BCL2	Body	0,008	-0,028	0,011	-0,051
cg02402914	BCL2	Body	-0,113	0,085	-0,037	0,281
cg07571261	BCL2	Body	-0,001	0,128	0,019	0,004
cg24168276	BCL2	Body	-0,131	0,232	0,008	0,165
cg12526942	BCL2	Body	-0,020	0,054	-0,091	0,045
cg24124688	BCL2	Body	0,014	0,025	-0,053	0,033
cg09330125	BCL2	Body	-0,066	0,115	-0,031	0,160
cg20221293	BCL2	Body	-0,014	0,181	-0,022	0,005
cg13580052	BCL2	Body	-0,072	0,076	0,053	-0,006
cg08223235	BCL2	Body	-0,025	-0,262	-0,016	-0,045
cg12459502	BCL2	Body	-0,004	0,105	-0,012	-0,069
cg25059899	BCL2	Body	-0,073	0,049	-0,003	-0,013
cg12310881	BCL2	Body	-0,004	-0,001	0,047	0,132
cg18683060	BCL2	Body	-0,098	0,154	-0,018	0,324
cg02907384	BCL2	Body	-0,070	0,290	-0,159	0,341
cg17574442	BCL2	Body	-0,024	-0,049	-0,052	0,028
cg24763804	BCL2	Body	-0,026	0,325	0,036	0,336

cg20505444	BCL2	Body	-0,021	0,215	-0,200	0,054
cg10326130	BCL2	Body	0,038	0,276	0,052	0,096
cg15079347	BCL2	Body	-0,045	0,138	-0,005	0,276
cg04217152	BCL2	Body	-0,017	0,063	0,010	-0,093
cg15002683	BCL2	Body	-0,125	0,241	-0,295	0,384
cg26433266	BCL2	Body	-0,142	0,357	-0,025	0,294
cg25373630	BCL2	Body	-0,076	0,137	-0,138	0,036
cg14544831	BCL2;BCL2	Body;3'UTR	0,014	-0,004	-0,024	-0,010
cg21602520	BCL2;BCL2	Body;Body	-0,082	0,004	-0,126	-0,028
cg14889643	BCL2;BCL2	Body;Body	-0,050	0,029	-0,060	-0,009
cg17602451	BCL2;BCL2	Body;Body	-0,048	0,011	-0,063	-0,020
cg24740531	BCL2;BCL2	Body;Body	0,072	-0,062	-0,032	0,013
cg05858920	BCL2;BCL2	5'UTR;5'UTR	-0,010	0,002	-0,049	-0,005
cg01803238	BCL2;BCL2	5'UTR;5'UTR	-0,006	-0,016	-0,039	-0,029
cg11330108	BCL2;BCL2	5'UTR;5'UTR	0,005	-0,007	-0,023	-0,027
cg27388618	BCL2;BCL2;BCL2;BCL2	1stExon;5'UTR;1stExon;5'UTR	0,003	-0,012	-0,018	-0,019
cg21777986	BCL2;BCL2;BCL2;BCL2	1stExon;5'UTR;1stExon;5'UTR	0,039	-0,024	-0,002	-0,073
cg09752703	BCL2;BCL2	TSS200;TSS200	-0,010	0,003	-0,066	0,015
cg12461368	BCL2;BCL2	TSS200;TSS200	-0,065	0,009	-0,016	-0,011
cg03813215	BCL2;BCL2	TSS200;TSS200	-0,012	-0,012	-0,018	-0,016
cg18423859	BCL2;BCL2	TSS200;TSS200	-0,031	-0,007	-0,054	0,008

cg05877143	BCL2;BCL2	TSS200;TSS200	-0,055	-0,007	-0,085	0,002
cg14604802	BCL2;BCL2	TSS200;TSS200	-0,053	0,045	-0,059	0,005
cg05945943	BCL2;BCL2	TSS200;TSS200	-0,071	0,041	0,019	-0,076
cg16445842	BCL2;BCL2	TSS1500;TSS1500	-0,027	0,016	-0,009	-0,007
cg17173732	BCL2;BCL2	TSS1500;TSS1500	-0,046	0,022	-0,007	-0,005
cg08554462	BCL2;BCL2	TSS1500;TSS1500	-0,055	0,012	-0,065	0,006
cg23699933	BCL2;BCL2	TSS1500;TSS1500	-0,039	-0,014	-0,050	-0,018
cg25730573	BCL2;BCL2	TSS1500;TSS1500	0,023	-0,006	0,023	-0,008
cg14455307	BCL2;BCL2	TSS1500;TSS1500	-0,064	0,020	-0,087	0,018
cg06881186	BCL2;BCL2	TSS1500;TSS1500	-0,023	-0,019	-0,019	-0,001
cg16003390	BCL2;BCL2	TSS1500;TSS1500	0,019	-0,010	0,017	-0,001
cg11255230	BCL2;BCL2	TSS1500;TSS1500	0,064	-0,056	0,000	-0,037
cg20777956	BCL2;BCL2	TSS1500;TSS1500	-0,037	0,043	-0,076	0,029
cg22152050	BCL2;BCL2	TSS1500;TSS1500	-0,038	0,063	-0,055	0,008
cg12953669	BCL2;BCL2	TSS1500;TSS1500	0,001	-0,018	-0,088	0,049
cg05724997	BCL2;BCL2	TSS1500;TSS1500	-0,005	-0,039	-0,102	0,042
cg12090740	BCL2;BCL2	TSS1500;TSS1500	0,019	-0,036	-0,107	0,031
cg26277730	BCL2;BCL2	TSS1500;TSS1500	-0,059	0,024	-0,080	0,019
cg21793948	BCL2;BCL2	TSS1500;TSS1500	-0,091	0,054	-0,117	0,059
cg23707289	BCL2;BCL2	TSS1500;TSS1500	-0,052	0,015	-0,068	0,009
cg26118326	MCL1;MCL1	3'UTR;3'UTR	-0,053	0,133	0,041	0,012

cg24998145	MCL1;MCL1	Body;Body	0,008	-0,035	-0,021	-0,049
cg06832064	MCL1;MCL1	Body;Body	-0,008	-0,012	-0,033	-0,027
cg00374009	MCL1;MCL1;MCL1;MCL1	Body;ExonBnd;1stExon;1stExon	-0,001	-0,009	-0,046	0,016
cg27166593	MCL1;MCL1;MCL1;MCL1	Body;ExonBnd;1stExon;1stExon	0,032	-0,025	-0,014	-0,011
cg10858523	MCL1;MCL1	1stExon;1stExon	0,001	-0,020	-0,004	-0,012
cg18397157	MCL1;MCL1	1stExon;1stExon	0,028	-0,030	0,030	-0,022
cg04228104	MCL1;MCL1	1stExon;1stExon	0,031	-0,039	0,153	-0,165
cg07561848	MCL1;MCL1	1stExon;1stExon	0,091	-0,084	0,062	-0,061
cg09804220	MCL1;MCL1	1stExon;1stExon	0,059	-0,047	0,014	-0,024
cg09726469	MCL1;MCL1;MCL1;MCL1	1stExon;1stExon;5'UTR;5'UTR	0,031	-0,035	-0,001	-0,035
cg17217603	MCL1;MCL1;MCL1;MCL1	1stExon;1stExon;5'UTR;5'UTR	0,048	-0,054	0,077	-0,088
cg16431914	MCL1;MCL1;MCL1;MCL1	1stExon;1stExon;5'UTR;5'UTR	-0,014	-0,014	-0,099	0,013
cg05431687	MCL1;MCL1;MCL1;MCL1;MCL1;MCL1	1stExon;1stExon;1stExon;5'UTR;5'UTR;5'UTR	-0,122	-0,010	-0,146	-0,036
cg24285922	MCL1;MCL1;MCL1;MCL1;MCL1;MCL1	1stExon;1stExon;1stExon;5'UTR;5'UTR;5'UTR	-0,021	-0,029	-0,132	-0,011
cg06217312	MCL1;MCL1	TSS1500;TSS1500	0,024	-0,025	0,017	-0,048
cg22649068	MCL1;MCL1	TSS1500;TSS1500	0,000	-0,031	-0,064	-0,041
cg13175981	MCL1;MCL1	TSS1500;TSS1500	0,004	-0,031	-0,063	-0,005
cg25183744	MCL1;MCL1;MCL1	TSS200;TSS200;TSS200	0,024	-0,025	-0,039	-0,030
cg27483771	MCL1;MCL1;MCL1	TSS1500;TSS1500;TSS1500	-0,002	-0,015	-0,018	-0,033
cg02961109	MCL1;MCL1	TSS1500;TSS1500	0,002	0,057	-0,064	0,113
cg18016565	MCL1;MCL1	TSS1500;TSS1500	-0,060	0,107	-0,018	0,055

cg25291404	BCL2L1;BCL2L1	3'UTR;3'UTR	0,013	-0,124	-0,009	0,239
cg08466983	BCL2L1;BCL2L1	Body;Body	0,004	-0,06	0,077	-0,057
cg21948170	BCL2L1;BCL2L1	Body;Body	0,026	-0,036	-0,089	-0,072
cg15488794	BCL2L1;BCL2L1	Body;Body	0,015	0,138	-0,271	0,422
cg07804698	BCL2L1;BCL2L1	Body;Body	0,064	-0,02	-0,083	-0,027
cg00013684	BCL2L1;BCL2L1	Body;Body	-0,016	0,001	-0,296	-0,017
cg01947399	BCL2L1;BCL2L1	Body;Body	-0,031	0,007	-0,003	-0,157
cg06892281	BCL2L1;BCL2L1	Body;Body	-0,051	0,171	-0,131	0,411
cg02538009	BCL2L1;BCL2L1	Body;Body	-0,009	-0,069	0,002	0,245
cg14518385	BCL2L1;BCL2L1	Body;Body	-0,023	0,02	-0,125	-0,102
cg14517873	BCL2L1;BCL2L1	Body;Body	-0,052	0,256	0,007	-0,042
cg17168956	BCL2L1;BCL2L1	Body;Body	-0,04	0,045	-0,057	0,134
cg11370496	BCL2L1;BCL2L1	Body;Body	0,067	-0,364	-0,019	0,144
cg04897370	BCL2L1;BCL2L1	Body;Body	-0,007	0,108	-0,025	0,361
cg21276232	BCL2L1;BCL2L1	Body;Body	0,021	0,047	-0,039	0,022
cg11265696	BCL2L1;BCL2L1	Body;Body	-0,016	0,005	-0,016	-0,032
cg04368572	BCL2L1;BCL2L1	Body;Body	-0,063	0,292	0,058	0,21
cg00300298	BCL2L1;BCL2L1	Body;Body	-0,054	0,118	-0,032	0,094
cg18787420	BCL2L1;BCL2L1	Body;Body	-0,029	0,042	-0,059	0,009
cg12873919	BCL2L1;BCL2L1	Body;Body	-0,029	0,094	0,038	-0,429
cg13989999	BCL2L1;BCL2L1	Body;Body	-0,043	0,13	0,037	-0,088

cg16137862	BCL2L1;BCL2L1	5'UTR;5'UTR	-0,133	0,213	0,121	-0,296
cg07602173	BCL2L1;BCL2L1;ABALON;BCL2L1;BCL2L1	1stExon;1stExon;Body;5'UTR;5'UTR	-0,089	0,039	-0,066	-0,042
cg21306641	BCL2L1;BCL2L1;ABALON;BCL2L1;BCL2L1	1stExon;1stExon;Body;5'UTR;5'UTR	-0,003	-0,029	0,03	-0,048
cg23509889	BCL2L1;BCL2L1;BCL2L1;BCL2L1	5'UTR;1stExon;5'UTR;1stExon	-0,028	-0,016	-0,013	-0,018
cg15025647	BCL2L1;BCL2L1;BCL2L1;BCL2L1	5'UTR;1stExon;5'UTR;1stExon	0,015	-0,026	-0,052	-0,026
cg11659435	BCL2L1;BCL2L1	TSS200;TSS200	-0,011	-0,008	0,043	-0,055
cg02457826	BCL2L1;BCL2L1	TSS200;TSS200	0,061	-0,066	0,077	-0,116
cg07379251	BCL2L1;BCL2L1	TSS200;TSS200	-0,103	0,075	-0,022	-0,033
cg00782854	BCL2L1;BCL2L1	TSS1500;TSS1500	-0,042	0	-0,084	0,003
cg12896779	BCL2L1;BCL2L1;ABALON	TSS1500;TSS1500;Body	-0,015	-0,01	-0,095	-0,007
cg11551419	BCL2L1;BCL2L1	TSS1500;TSS1500	-0,009	-0,006	-0,04	-0,012
cg08619561	BCL2L1;BCL2L1	TSS1500;TSS1500	-0,006	-0,039	-0,02	-0,016
cg11809604	BCL2L1;BCL2L1	TSS1500;TSS1500	0,019	-0,024	0,001	-0,031
cg23752198	BCL2L1;BCL2L1	TSS1500;TSS1500	-0,003	-0,001	0,047	-0,062
cg00058652	ABALON;BCL2L1;BCL2L1	TSS1500;Body;Body	0,05	-0,025	-0,122	-0,063
cg09190106	ABALON;BCL2L1;BCL2L1	Body;5'UTR;5'UTR	0,036	-0,034	0,011	-0,04

Table S5.

Overview of the name, drug class and mechanism of action of all 13 compounds used in the drug-screen experiments.

**Compound**	**Drug class**	**Specific mechanism og action**
Vorinostat		HDAC inhibitor
Tazemetostat (EPZ-6438)		EZH2 inhibitor
Valemetostat (DS-3201b)	Epigenetic drugs	EZH1/EZH2 inhibitor
Metformin	Metabolic modifier	AMPK activator
Selinexor (KPT-330)	Nuclear transport inhibitor	XPO1 inhibitor
Copanlisib (BAY 80-6946)		pan-class I PI3K inhibitor
Everolimus		mTOR1 inhibitor
Sapanisertib (TAK-228)		mTOR1/mTOR2 inhibitor
Gedatolisib (PKI-587)		PI3Kα, PI3Kγ, and mTOR inhibitor
TIC10 (ONC-201)		Akt and ERK inhibitor
Capivasertib (AZD5363)		Akt inhibitor
Idelalisib (CAL-101)	PI3K/Akt/mTOR inhibitors	p110δ inhibitor
IQ3	Interleukin inhibitor	c-Jun N-terminal kinase (JNK) inhibitor
